# Temporal Changes in Concentrations of Some Trace Elements in Muscle Tissue of Crayfish, *Astacus leptodactylus* (Eschscholtz, 1823), from Keban Dam Lake

**DOI:** 10.1155/2014/120401

**Published:** 2014-02-23

**Authors:** Onder Aksu, Ragip Adiguzel, Veysel Demir, Numan Yildirim, Durali Danabas, Sebahat Seker, Safak Seyhaneyildiz Can, Mustafa Ates

**Affiliations:** ^1^Fisheries Faculty, University of Tunceli, 62000 Tunceli, Turkey; ^2^Department of Chemical Engineering, Faculty of Engineering, University of Tunceli, 62000 Tunceli, Turkey; ^3^Department of Bioengineering, Faculty of Engineering, University of Tunceli, 62000 Tunceli, Turkey; ^4^Department of Environmental Engineering, Faculty of Engineering, University of Tunceli, 62000 Tunceli, Turkey; ^5^Department of Environmental Engineering, Faculty of Engineering, University of Ardahan, 75000 Ardahan, Turkey; ^6^Vocational School of Tunceli, University of Tunceli, 62000 Tunceli, Turkey

## Abstract

Crayfish (*Astacus leptodactylus* Eschscholtz, 1823) is the native crayfish species in Turkey. It was exported regularly to Western Europe. In this study, bioaccumulation and temporal trends of some trace elements (arsenic: As, cadmium: Cd, copper: Cu, mercury: Hg, lead: Pb, and zinc: Zn) in edible abdomen muscle of crayfish from Keban Dam Lake (Elazığ, Turkey) were investigated for the 2006–2012 period. Sequence of metal concentration levels was Zn > Cu > Hg > Pb > Cd > As in muscle tissues. The highest concentration of Zn (21.69 mg kg^−1^) was detected in 2006, while the lowest (4.35 mg kg^−1^) in 2009. In general, it was found that the concentrations of trace elements investigated were lower than the maximum permissible limits of the food regulations of the Ministry of Food, Agriculture, and Livestock (MFAL), the Turkish Food Codex and Commission Regulation (EC). If the crayfish selected for the study are recognized as bioindicators of environmental pollution, then it is possible to conclude that the changes in studied trace elements concentrations in the Keban Dam Lake are being steady.

## 1. Introduction

Many pollutants including trace elements are released from natural and anthropogenic sources into aquatic environments [1, 2]. Accumulation of these elements in sediments, aquatic biota, and edible aquatic organisms is an important concern, because they are easily involved in food chain and affect most important reactions in living organisms, even at low concentrations [1, 3, 4].

Copper (Cu) can enter the environment through releases from the mining of Cu and other metals and from factories that make or use Cu metal or Cu compounds. Copper can also enter the environment through waste dumps, domestic waste water, combustion of fossil fuels and wastes, wood production, phosphate fertilizer production, and natural sources. One of the most commonly reported adverse health effect of Cu is gastrointestinal distress [5].

Cadmium (Cd) is emitted to soil, water, and air by nonferrous metal mining and refining, manufacture and application of phosphate fertilizers, fossil fuel combustion, and waste incineration and disposal. Cadmium can accumulate in aquatic organisms and agricultural crops. The U.S. Department of Health and Human Services (DHHS) has determined that Cd and Cd compounds are known human carcinogens [6].

Lead (Pb) and Pb alloys are commonly found in pipes, storage batteries, weights, shot and ammunition, cable covers, and sheets used to shield us from radiation. The largest use for lead is in storage batteries in cars and other vehicles. Lead compounds are used as a pigment in paints, dyes, and ceramic glazes and in caulk. The amount of Pb used in these products has been reduced in recent years to minimize Pb's harmful effect on people and animals. Lead has long been known to alter the hematological system by inhibiting the activities of several enzymes involved in heme biosynthesis [7].

Mercury (Hg) is a naturally occurring metal and enters the environment as the result of the normal breakdown of minerals in rocks and soil from exposure to wind and water, and from volcanic activity. Approximately 80% of the Hg released from human activities are elemental Hg released to the air, primarily from fossil fuel combustion, mining, and smelting and from solid waste incineration. About 15% of the total is released to the soil from fertilizers, fungicides, and municipal solid waste (e.g., from waste that contains discarded batteries, electrical switches, or thermometers). An additional 5% is released from industrial wastewater to water in the environment [8].

Zinc (Zn) is an essential human nutrient and a cofactor for over 300 enzymes and is found in all tissues. In humans, the highest concentrations of Zn have been found in bone, muscle, prostate, liver, and kidneys [9, 10]. Large oral doses of Zn can interfere with Cu bioavailability as they compete for absorption, and clinical signs of immune dysfunction have been reported with daily doses in excess of 150 mg [11].

The most essential component of life, water, being contaminated with arsenic is a global human health hazard. Millions of the populations worldwide are exposed to arsenic-contaminated drinking water. Arsenic (As) is widely distributed in nature and released into the environment through natural sources, industrial processes, and agriculture usage [12]. Arsenic can affect human health and is considered one of the most significant environmental causes of cancer in the world [13]. Reference [14] reported that As is a unique carcinogen. It is the only known human carcinogen for which there is adequate evidence of carcinogenic risk by both inhalation and ingestion.

Some trace elements including Zn and Cu which are taken up by aquatic invertebrates both from food and solution are known to be essential elements and play important roles in biological metabolism at very low concentrations [15–18].

Others are known to be toxic, even at low concentrations, including aluminum (Al), As, Cd, chromium (Cr), iron (Fe), manganese (Mn), Hg, nickel (Ni), selenium (Se), Zn, and Pb [15, 19]. These elements can cause adverse effects on aquatic organisms, if these chemicals exist in biota and eventually build up to unacceptable levels in these organisms. Some trace elements are major contributor to crucial biochemical reactions in many living organisms such as fish and crustaceans [1, 20].

Generally crustaceans like all aquatic invertebrates accumulate metals from a wide range of sources and the trace metal concentrations within their tissues and bodies show great variability [21]. Crayfish can be used to monitor the aquatic environments for heavy metal pollution because they are solitary bottom dwellers, which keep much of their bodies in contact with surrounding objects and tend to accumulate metals in their tissues [22, 23]. These crayfish are used as a vector of contamination in many studies [3, 21, 22, 24, 25].

Due to negative effect of trace elements, their bioaccumulation in edible tissues of aquatic invertebrates needs to be monitored. The objective of this study is to determine temporally the bioaccumulation of trace elements in abdomen muscles of crayfish, *Astacus leptodactylus, *which is sea food having a great attraction in human consumption.

## 2. Materials and Methods

### 2.1. Properties of Sampling Area

**Figure 1:**
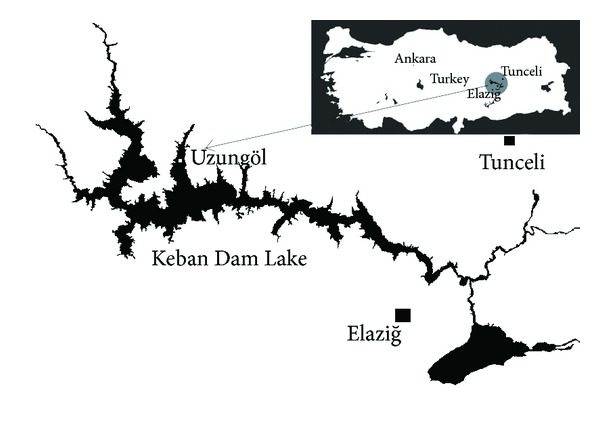
The map of sampling stations.

The Keban Dam Lake, which is a hydroelectric dam on the river Euphrates, is located between the cities of Elazığ and Tunceli. The surface area of Keban Dam Lake is 687.31 km^2^. It is situated at latitude 38°5′ N and 38°4′ E longitude at an elevation of 1134 m above sea level. This study was conducted in region with the sampling site coordinates of N 38° 57′ 09′′ E 38° 53′ 19′′ on Keban Dam Lake (Figure 1).

Near the sampling area, there are some metal processing industries that contribute to pollution of Keban Dam Lake. The effluent from Elazığ city sewage treatment facility is discharged to Keban Dam Lake. There are also some cage aquaculture facilities around sampling area.

### 2.2. Crayfish Sampling

The freshwater crayfish (*A. leptodactylus*) were caught by fyke nets in sampling area from Keban Dam Lake (Figure 1) each year in June and July from 2006 to 2012. The samples were sealed via plastic bags and transferred to the laboratory under cold chains. For each year, twenty five crayfish have approximately the same weight and length (same condition index) and were selected randomly among crayfish that is bigger than 10 cm, legal catching length, and the abdomen muscles of crayfish were dissected. The abdomen muscles of all crayfish were homogenized with tissue homogenizer and used for trace element determination.

### 2.3. Analysis

The concentrations of trace elements were measured with ICP MS (Perkin Elmer Germany) according to the methods (no. 161) described by NMKL [26] in Food Control Laboratory in Izmir (Turkey). The concentrations obtained from the analyses were compared with the same tissue (muscle) and species (crayfish) in MFAL and EC. The detectable limits of elements were 0.003, 0.001, 0.05, 0.01, 0.01, and 0.01 mg kg^−1^ for As, Hg, Zn, Cd, Cu, and Pb, respectively.

## 3. Results and Discussions

The concentrations of trace elements in abdomen muscles of crayfish were shown in Table 1. The highest Zn and Cu accumulation was measured as 21.69 and 7.2 mg kg^−1^, respectively, in 2006. There is a decrease in the concentration of both metals between 2006 and 2009 and an increase in following years. The levels of As were detected only in 2006 and 2011. The As, Cd, and Pb bioaccumulation levels were measured under the detectable limits for most of the samples analyzed. In general, there is an increase in mercury bioaccumulation level. The highest levels of Cu and Cd were determined in 2006 and 2008, while the lowest levels were determined in 2009. The antagonism between Cu and Cd was observed.

*A. leptodactylus* is the native crayfish species in Turkey. It was exported regularly to Western Europe, although domestic consumption of *A. leptodactylus* was very little. There has been an increase in the production of *A. leptodactylus* in recent years. One of the introduced populations of *A. leptodactylus* in Turkey is that in Keban Dam Lake, Elazığ [27].

Lake sediment represents an important sink for trace metals in aquatic systems, and metal concentrations in sediment can be several orders of magnitude greater than in the overlying water [28, 29]. Researchers determined some trace element concentration in water and sediment samples of Keban Dam Lake. Average Zn concentration was measured as 1.28 ppm in water samples and average concentration of Zn, Cu, Cr, Co, and Ni in sediment samples as 1473, 32.7, 198, 50, and 198 ppm, respectively [30].

In aquatic systems, crayfish have been a widespread importance to monitor metals and other contaminants [31], because of consuming widely and increasingly in human diets in the world [32]. Most crayfish are omnivorous benthic feeders living in close contact with the sediment and taking a variety of both animal and plant material [33, 34]. Few data are available on trace metal concentrations in benthic invertebrates from Keban Dam Lake, Elazığ, Turkey. In the present study, freshwater crayfish (*A. leptodactylus*) were used as the bioindicator organisms for monitoring and assessment of the water quality of the Keban Dam Lake for trace metals. Because they are closely associated with surficial sediments, and their effective bioaccumulation capacity of toxic metals has potential for transferring contaminants up the food web to human consumers.

In this study, the concentrations of trace elements determined were at permissible levels set by Turkish legislation in all years. No distinctive bioaccumulation trend was observed among years for any trace elements studied. Bioaccumulation of cooper, zinc, and mercury has a decreasing trend between 2006 and 2009 and then periods have showed increasing trend. But, for Hg and Pb, there is an increase in bioaccumulation level in last two years. That increased level can be associated with increasing cage aquaculture facilities and agricultural activities around sampling area. Similarly [35] reported that the aquaculture industries such as cage aquaculture facilities may discharge many chemical pollutants like trace elements and PAHs to aquatic environment especially due to the use of antibiotic, agrochemicals formulated feed.

## 4. Conclusion

In this study, the results of trace elements concentrations in *A. leptodactylus* which were captured from Keban Dam Lake between 2006 and 2012 were reported. Our results showed that bioaccumulation can occur in edible tissues. This study will provide significant data for further water quality monitoring studies to establish effects of trace element presence on living organisms in Keban Dam Lake area.

## Figures and Tables

**Table 1:** The levels of trace elements in abdomen muscles of crayfish (*A. leptodactylus* Eschscholtz, 1823) between 2006 and 2012 in Keban Dam Lake (Elazığ, Turkey).

Trace elements (mg kg^−1^)	Sampling periods						
	2006	2007	2008	2009	2010	2011	2012
As	0.037	UDL*	UDL	UDL	UDL	0.146	UDL
Cd	0.01	UDL	0.02	UDL	0.01	UDL	0.01
Cu	7.20	5.28	4.39	2.42	3.81	4.74	6.967
Hg	0.193	0.160	0.059	0.016	0.150	0.983	0.418
Pb	0.03	UDL	UDL	UDL	0.01	0.04	0.08
Zn	21.69	17.95	16.22	4.35	14.60	16.04	21.65

*UDL: under detection limit.
